# ^1^H, ^13^C and ^15^N chemical shift assignments of Na-FAR-1, a helix-rich fatty acid and retinol binding protein of the parasitic nematode *Necator americanus*

**DOI:** 10.1007/s12104-012-9444-4

**Published:** 2012-11-20

**Authors:** M. Florencia Rey-Burusco, Marina Ibañez-Shimabukuro, Alan Cooper, Malcolm W. Kennedy, Betina Córsico, Brian O. Smith

**Affiliations:** 1 Facultad de Ciencias Médicas, Instituto de Investigaciones Bioquímicas de La Plata, CONICET-UNLP, calles 60 y 120, 1900 La Plata, Argentina; 2 School of Chemistry, University of Glasgow, Glasgow, G12 8QQ UK; 3 Institute of Biodiversity, Animal Health and Comparative Medicine, University of Glasgow, Glasgow, G12 8QQ UK; 4 Institute of Molecular, Cell and Systems Biology, University of Glasgow, Glasgow, G12 8QQ UK

**Keywords:** Parasitic nematode, *Necator americanus*, Fatty-acid and retinol-binding protein, Na-FAR-1, NMR

## Abstract

The fatty acid and retinol-binding (FAR) proteins are a family of unusual helix-rich lipid binding proteins found exclusively in nematodes, and are secreted by a range of parasites of humans, animals and plants. Na-FAR-1 is from the parasitic nematode *Necator americanus,* an intestinal blood-feeding parasite of humans. Sequence-specific ^1^H, ^13^C and ^15^N resonance assignments have been obtained for the recombinant 170 amino acid protein, using three-dimensional triple-resonance heteronuclear magnetic resonance experiments. Backbone assignments have been obtained for 99.3 % of the non-proline HN/N pairs (146 out of 147). The amide resonance of T45 was not observed, probably due to rapid exchange with solvent water. A total of 96.9 % of backbone resonances were identified, while 97.7 % assignment of amino acid sidechain protons is complete. All Hα(166), Hβ(250) and Hγ(160) and 98.4 % of the Hδ (126 out of 128) atoms were assigned. In addition, 99.4 % Cα (154 out of 155) and 99.3 % Cβ (143 out of 144) resonances have been assigned. No resonances were observed for the NH_n_ groups of R93 N_ε_H_ε_, arginine, N_η1_H_2_, N_η2_H_2_, histidine N_δ1_H_δ1_, N_ε1_H_ε1_ and lysine N_ζ3_H_3_. Na-FAR-1 has a similar overall arrangement of α-helices to Ce-FAR-7 of the free-living *Caeorhabditis elegans,* but with an extra C-terminal helix.

## Biological context

Over 500 million people living in tropical and subtropical regions are infected with human hookworms, *Necator americanus* being the most prevalent. The infective larvae burrow into skin and develop to adulthood in the intestine, where they feed on blood and tissue. The infection causes anaemia and growth stunting, the effects being most severe in children and women of child bearing age (Hotez et al. 2004).

Fatty acid and retinol-binding (FAR) proteins are α-helix-rich proteins of around 20 kDa that are produced at different life cycle stages of nematodes and have been observed to be secreted by adult parasites (Basavaraju et al. 2003; Kennedy et al. 1997; Prior et al. 2001). It is hypothesized that FARs may play a role in host-parasite interaction and pathogenesis by sequestering or delivering lipid mediators, thereby affecting the local tissue environment of the host, and compromising immune and inflammatory defences (Bradley et al. 2001). FAR proteins are already used as diagnostic tools (Burbelo et al. 2009), and have also been shown to induce immunity against one parasite infection (Fairfax et al. 2009). They are, therefore, attractive potential targets for drug or vaccine development because they have no structural counterparts in mammals (Basavaraju et al. 2003).

Na-FAR-1 has been identified in a transcriptomic survey of *N. americanus* (Daub et al. 2000), and we here report resonance assignments of its bacterial recombinant form. The only structure of a FAR currently available is a crystal structure of Ce-FAR-7 of the free living nematode *Caenorhabditis elegans* (Jordanova et al. 2009). This FAR has seven α-helixes and comprises two binding sites that could accommodate different types of ligands. We find that Na-FAR-1 has a similar overall arrangement of regular secondary structure elements, but with an extra helical region at the C-terminus.

## Methods and experiments

Recombinant Na-FAR-1 was expressed in BL21 (λDE3) *Escherichia coli* cells using [^13^C, ^15^N]-labelled M9 minimal medium. The His-tagged fusion protein was purified by nickel affinity and gel filtration chromatographies. The removal of copurifying ligands from the bacterial expression system was achieved by reverse-phase high performance liquid chromatography (RP-HPLC) with a C8 stationary phase and water/acetonitrile/trifluoroacetic acid mobile phase. The protein was refolded in aqueous buffer and concentrated to approximately 0.5 mM in 50 mM sodium phosphate pH 7.2, 50 mM NaCl. D_2_O was added to a final concentration of 10 % (v/v) prior to data acquisition. All spectra were recorded at 311 K on a Bruker Avance 600 MHz spectrometer equipped with a TCI cryoprobe.

Sequence-specific resonance assignment of the Na-FAR-1 backbone was accomplished with the aid of 2D ^15^N-HSQC, 3D HNCACB, 3D CBCA(CO)NH (Muhandiram and Kay 1994), 3D HNCO (Kay et al. 1994) and 3D HNCACO spectra. The majority of aliphatic sidechain carbon and proton resonances were located by navigating from the backbone data using 3D (H)C(CO)NH-TOCSY, 3D HBHA(CBCA)NH (Wang et al. 1994) and 3D H(C)(CO)NH-TOCSY spectra (Grzesiek and Bax 1992). Remaining aliphatic resonances were identified using 3D HcCH-TOCSY and 3D hCCH-TOCSY (Kay et al. 1993), this latter proving particularly useful for assignment of lysine sidechain C_δ_H_δ_ and C_ε_H_ε_ groups that were too overlapped in both ^1^H and ^13^C dimensions to be resolved in other experiments. A proportion of aromatic sidechain ^13^C/^1^H signals (histidine H_δ1_, tyrosine H_δ,ε_ and phenylalanine H_δ,ε_) were assigned using 2D HBCBCGCDHD and 2D HBCBCGCDCEHE spectra (Yamazaki et al. 1993) and the remainder were identified from the ^13^C-edited [^1^H,^1^H]-NOESY spectrum. NMR spectra were processed using AZARA (http://www.bio.cam.ac.uk/azara) and analysed with CCPN analysis (Vranken et al. 2005).

## Extent of assignments and data deposition

**Fig. 1:**
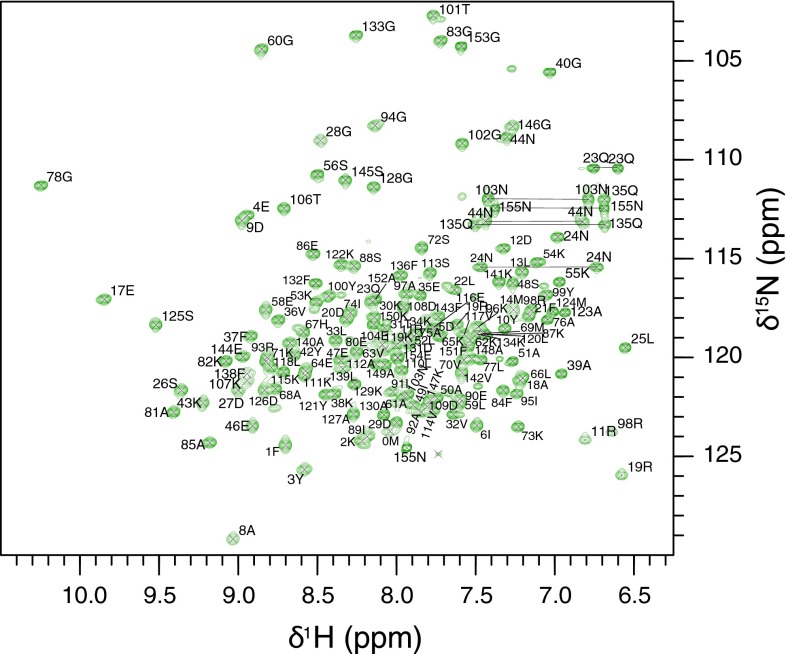
Two-dimensional ^1^H–^15^N HSQC spectrum of recombinant Na-FAR-1 showing the backbone amide resonance assignments. *Crosspeaks* have been labelled with the *single letter* amino acid code along with the native sequence specific number

All Na-FAR-1 polypeptide backbone resonances were assigned, with the exception of the 15 His-tag *N*-terminal residues (MGSSHHHHHHSSGHM). Excluding the His-tag residues, backbone resonance assignments have been obtained for 99.3 % of the non-proline HN/N pairs (146 out of 147). The amide resonance of T45 was not observed, probably due to rapid exchange with solvent water. Figure 1 shows the ^1^H–^15^N HSQC spectrum with the assigned crosspeaks. A total of 96.9 % of backbone resonances were identified, while 97.7 % assignment of amino acid sidechain protons is complete. All Hα(166), Hβ(250) and Hγ(160) and 98.4 % of the Hδ (126 out of 128) atoms were assigned. In addition, 99.4 % Cα (154 out of 155) and 99.3 % Cβ (143 out of 144) resonances have been assigned. No resonances were observed for the following NH_n_ groups: R93 N_ε_H_ε_, arginine, N_η1_H_2_, N_η2_H_2_, histidine N_δ1_H_δ1_, N_ε1_H_ε1_ and lysine N_ζ3_H_3_.

**Fig. 2:**
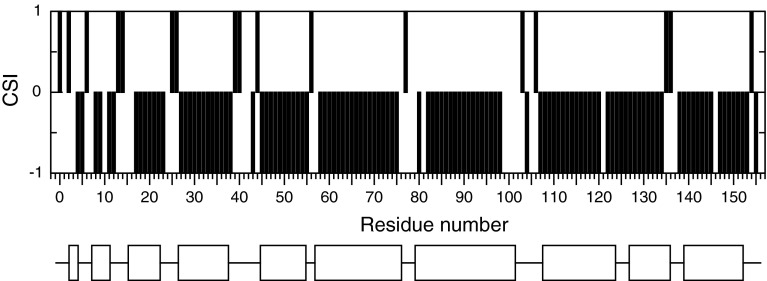
Na-FAR-1 CSI consensus values (*bars*) and secondary structure elements (*bottom*) alignment. Negative CSI indicate α-helical segments which are shown as *boxes* below

Chemical shift index (CSI) (Wishart and Sykes 1994) and DANGLE secondary structure analysis (Cheung et al. 2010) of Na-FAR-1 revealed a α-helix pattern consistent with the previously reported *C. elegans* FAR protein, with an additional α-helix segment at the C-terminus (Fig. 2).

The ^1^H, ^13^C and ^15^N chemical shift assignments have been deposited with the BioMagResBank database (http://www.bmrb.wisc.edu), accession number 18637.
